# State of the Patients in the Bristol Dispensary, from 1st of April to the 1st of May, 1801

**Published:** 1801-06

**Authors:** 


					State of the Patients in the Brifiol Difpenfary, from \Jl of April
'to the \Ji cf May, 1801.
1 yphus r ever - - - -51
Continued Fever - - 5
Dropfy - - - - - 10
Tabes - - ^ - - - - 6
Paralylis - - - - _ j
Stomach Complaints ? - 7
Amenorrhoea - - - 1
Afthenia - - - - - 2
Peripneumonia - - - 8
Scrophula - 3
Diarrhoea and Dyfentery - 7
omall-rox - - - - 2
Cough and Dyfpncea - - .3
Rheumatifm z
Haemoptoe ----- 1
Eryiipelas - - ' - 3
Nervous Affe&ions - - 2
Afthma 3
Pleuritic AfFedtions - - 2
Hernia ----- - 1
Bilious Vomiting - - - - 2
Phthifis Pulmona.is - - 2

				

